# Disturbance gradient and mosquito diversity pattern in areas surrounding Chini Lake - the second largest freshwater lake in Peninsular Malaysia

**DOI:** 10.3897/BDJ.10.e83800

**Published:** 2022-07-07

**Authors:** Taneswarry Sethu Pathy, Jin Min Lee, Sze Huei Yek

**Affiliations:** 1 School of Science, Monash University Malaysia, Bandar Sunway, Malaysia School of Science, Monash University Malaysia Bandar Sunway Malaysia; 2 Institute for Tropical Biology and Conservation, Universiti Malaysia Sabah, Kota Kinabalu, Malaysia Institute for Tropical Biology and Conservation, Universiti Malaysia Sabah Kota Kinabalu Malaysia

**Keywords:** anthropogenic disturbance, larval breeding grounds, land-use changes, host availability, *Aedes* spp.

## Abstract

Malaysia is a tropical country that has consistently been facing a prevalent threat of mosquito-borne diseases. Amongst the plethora of diseases, the most common mosquito-borne disease in the country is dengue fever, transmitted by *Aedes* spp. mosquitoes. This study aims to examine the effects of human activity associated with different land use on mosquito population composition and diversity. Our study site is Chini Lake, a naturally occurring lake and the second-largest freshwater body in Malaysia. The areas surrounding the Lake have been subjected to various human activities, such as economic development and conversion into rubber plantations, while some areas remain pristine, making Chini Lake an ideal location to examine the gradient of human disturbances on mosquito composition and diversity. We sampled adult mosquitoes and larvae across a range of sites with different levels of human disturbance. As expected, in areas with high disturbance scores, the species richness of adult and larval mosquitoes were reduced while the abundance was higher. The results also revealed minimal overlap between species captured for adult and larval samplings, suggesting that land-use changes affect both life stages differently. Different resource requirements of adult and larval mosquitoes likely led to the observed diversity pattern in this small survey. We suggest future work to look into how habitat heterogeneity affects both life stages and sexes of mosquito diversity patterns and distribution.

## Introduction

Mosquito-borne diseases are mainly concentrated in tropical and sub-tropical regions of the world and are a source of global health concern (Liang et al. 2019). Malaysia is particularly susceptible to mosquito-borne diseases as the wet tropical climate is ideal for mosquitoes to thrive in, with the wet season seeing outbreaks of such diseases (Lafferty 2009). The most common and widely-spread mosquito-borne disease in Malaysia is dengue fever, vectored by the *Aedes* spp. mosquitoes. Other mosquito-borne diseases in Malaysia include chikungunya, Japanese encephalitis and malaria (Shafie et al. 2016). There is an increasing pattern of dengue incidences throughout the entire country for the past decade, with west peninsular states affected more severely by the dengue fever. These incidences were concentrated in urban communities (Mohd-Zaki et al. 2014), suggesting that there is a positive correlation between the prevalence of dengue fever and the increased development of urban landscapes. The year-long chikungunya outbreak in Malaysia was also heavily concentrated in urbanised environments (Lam et al. 2001), further reinstating that the incidence of mosquito-borne diseases in Malaysia is associated with increased urbanisation.

Human activities, such as deforestation and agricultural development, have been associated with the increased transmission of arboviruses by mosquitoes (Vasconcelos et al. 2001, Norris 2004). Changes in land use across various natural mosquito habitats act as a driving force that alters the behaviour of mosquitoes as they source alternative habitats and resources (Norris 2004, Lee et al. 2019), which directly interrupts the regular host-seeking behaviour of blood-feeding mosquitoes due to the changes in host availability as the landscape is transformed (Zahouli et al. 2017b). Deforestation is a key contributor to the destruction of natural mosquito habitats and subsequent behavioural alterations of mosquitoes (Afrane et al. 2006, Olson et al. 2010). Forests that are rich in flora and fauna provide shelter and an abundance of food resources for blood-feeding and non-blood-feeding mosquitoes alike. When such forests are destroyed, mosquitoes are forced to alter their behaviour in response to the disturbances. These changes in feeding habits of mosquitoes contribute to the increased risk of mosquito-borne disease transmission amongst humans, potentially explaining the increase in mosquito-borne diseases in urbanised environments (Olson et al. 2010, Zahouli et al. 2017a).

While natural mosquito habitats are destroyed by changes in land use, certain human activities may provide an abundance of breeding opportunities for certain mosquito species, especially container breeders and species that can thrive in feculent waters (Li et al. 2014, Zahouli et al. 2017a, Zahouli et al. 2017b, Lee et al. 2019). Human activities that result in the introduction of artificial containers and fruit husks can act as driving factors in breeding of such mosquito species (Li et al. 2014). An example of this would be the transformation of forests into rubber plantations where containers and fruit husks are introduced to collect latex from rubber trees. These containers can act as breeding grounds for container-breeders (Rao 2010, Tangena et al. 2016). Container breeders, such as the *Aedes* and *Culex* mosquito, can exploit these introduced artificial containers and displace other mosquito species in a given area (Li et al. 2014). The ability of the *Aedes* and *Culex* mosquito larvae to survive in such containers under constrained circumstances gives them a competitive edge against other mosquito species that fail to display similar behaviour, resulting in competitive exclusion of other species in terms of breeding grounds (Miyagi et al. 1992, Koenraadt and Harrington 2008).

**Figure 1.**
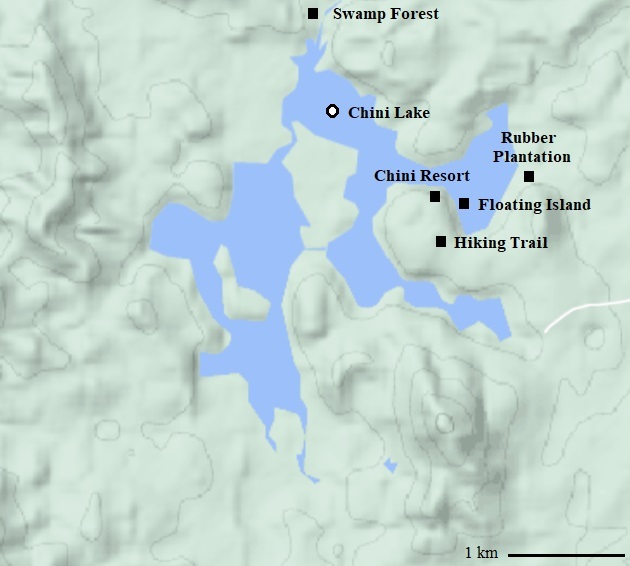
Five sampling sites (black squares) within a one-kilometre radius of Chini Lake. The sampling sites are situated at an accessible section of the Lake. The light blue denotes the water body of Chini Lake and the different shades of green denote the contour of the land around the Lake.

Chini Lake - the second largest freshwater lake in Malaysia - is under threat from human development (Sharip and Jusoh 2010). The Lake is surrounded by hilly areas and undulating land and is comprised of 202 ha of open water and 700 ha of swamp area (Plan 1987). The Lake is unusual in shape, comprising 12 small lakes interconnected by natural channels and is categorised as an alluvial riparian swamp system, an ecosystem that is categorised as the most endangered ecosystem type in Malaysia (Hebauer et al. 1999; Fig. 1). The lake has recreational value as well as a hub for its unique biodiversity. It possesses natural landscapes and social-cultural attractions, with surrounding areas primarily inhabited by the aboriginal Jakun people and local Malay Federal Land Development Authority (FELDA) settlers (Sujaul et al. 2010). The aboriginal people and local Malay settlers carry out daily activities, such as collecting rattan, bamboo, wood, resin, roots and medicinal plants, hunting and fishing. They also engage in agricultural activities, such as rubber tapping, as some forests close to their settlement have been converted into rubber plantations (Kemalok et al. 2019). Intense mining activities have also been conducted by private corporations in the areas surrounding the Lake, where forests have been cleared, contributing to the pollution of the Lake (Sujaul et al. 2010). This clearing has also led to indiscriminate piling of soil and plant debris around the Lake, which over time, led to the colonisation of pioneer plants and subsequent flooding (Sharip and Jusoh 2010). During the flooding period, these piles can be dislodged from the Lake’s littoral zone and move around the Lake, which led to the formation of floating islands on the Lake. These understudied floating islands present a unique habitat as they are situated on the Lake itself and do not house any large mammals (Sujaul et al. 2010, Sharip and Jusoh 2010).

Despite such a wide range of land use in Chini Lake, Pahang, the effects of human activity on mosquito composition and diversity remain underexplored. Dengue cases have continuously risen in the State of Pahang over the decade (Syafiq Mustafa and Sulaiman 2021). Besides the dengue fever, cases of malaria, another mosquito-borne disease carried by the *Anopheles* mosquito, were also reported in the rural areas of the State of Pahang (Kaur 2009, Ahmad et al. 2011). Although mosquito-borne diseases have been reported across the State of Pahang, studies regarding how the changes in land use affect mosquito composition and diversity, which provides insightful information on mosquito-borne disease transmission, have not been previously carried out in this area. We contend that the terrestrial environment surrounding the Lake was an ideal study location because of its mix of pristine, non-human-disturbed areas and areas with high human traffic, representing a disturbance gradient to be studied concerning mosquito composition and diversity. Therefore, this study will assess: (i) the effects of a human disturbance gradient on mosquito species richness and abundance in five different areas around Lake Chini. Additionally, as mosquitoes have dual life stages with aquatic larval form and adult terrestrial form, we will also assess: (ii) the impact of the human disturbance on these two life stages separately. Furthermore, we will look at: (iii) the community composition of mosquitoes at the different sites. We hypothesise that mosquito species richness will decrease, whereas mosquito abundances will increase with increasing human disturbance. From existing works of literature, we also expect to find low community composition overlap between disturbed and pristine sites.

## Material and methods

### Study area and quantifying disturbance scale

This study was conducted in the east coast region of Malaysia. The chosen study site, Chini Lake, is located in the State of Pahang. The five sampling sites were approximately within a one-kilometre radius of Chini Lake (33°26’N, 102°55’E; Fig. 1), where the sampling sites ranged from areas with high anthropogenic disturbance to pristine areas with low anthropogenic disturbance. Human disturbance was quantified according to the disturbance scale presented by Kimberling et al. (2001). Four distinct measures were considered when assessing the level of anthropogenic disturbance in a given sampling site. These four measures were: the extent of disturbance, the extent of soil profile disturbance, time since disturbance and frequency of disturbance. Scores ranged from 0-2, at 0.5 increments, whereby 0 represents low disturbance and 2 represents high disturbance. The scores for each scale were given, based on observations by two independent researchers' (TSP and JML) assessments at the sampling sites, with the tallying of scores across measures used as the overall disturbance score per site. In situations where the scoring differed between the assessors, we took the average as the disturbance score (Table 1). Both independent researchers scored Chini Resort as the site with the highest anthropogenic disturbance, followed by the rubber plantation with moderate anthropogenic disturbance. It is located in a village of the Jakun aboriginal people and is mainly utilised by the aboriginal people for rubber-tapping activities. A hiking trail in a secondary forest is also scored as a site with a moderate anthropogenic disturbance with aboriginal people using this trail for collecting natural products, such as wood, resin and hunting wildlife in the forest. The floating island is a unique sampling site because it is mobile during the flood season and has limited accessibility as it can only be accessible by boat and has no economic value to the tourists or the local community. However, due to the nature of the floating island, the researchers scored a 1.0 for the category 'time since disturbance'. An alluvial riparian swamp with limited accessibility to tourists and the local community represents the site with no anthropogenic disturbance.

### Mosquito sampling and species identification

The sampling of mosquitoes was conducted between 3 and 5 July 2018 at five different sites within the study area, which coincides with the dry season in Pahang (Fig. 1). These sites are only accessible during the dry season as the roads accessing the Chini Lake areas will be flooded during the wet season. For adult mosquito sampling, each site was sampled twice for 30 minutes respectively, once in the morning and once in the evening, within the periods of 7:00 AM to 8:00 AM and 6:00 PM to 7:00 PM. We chose the mosquito sampling timing to coincide with known peak female mosquito foraging activity. A sampling focal point was chosen in each of the five sampling sites and sampling was done within a 100-m radius of that point. Using the Prokopack Aspirator Model 1419 (JW Hock, USA), adult mosquitoes were captured by targeting potential mosquito resting places and any visible mosquito at ground level to a height of 2 m - the maximum height reach of the Prokopack Aspirator. Captured mosquitoes were then stored in sealable plastic bags to be transported back to the lab. Mosquito samples were transferred into a killing jar filled with 75% ethanol for identification purposes.

For mosquito larval sampling, we surveyed within the 100 m sampling focal point from each of the five sampling sites (Fig. 1). Larvae were collected from all available larvae habitats, natural and/or artificial, found within this radius at each sampling site. Larval habitats that were made up of small water bodies were emptied into a plastic container where mosquito larvae were subsequently picked out using a plastic pipette or directly picked out from their habitat. Larval habitats that were made up of larger water bodies were pumped using a hand siphon pump, where the water was collected in plastic containers and the mosquito larvae were subsequently picked out using a plastic pipette. Collected mosquito larvae were kept alive and transported back to the laboratory where they were reared until adults for identification. The mosquito identification was based on the morphological distinctions of mostly female adult mosquitoes under a microscope using several taxonomic keys developed for Thailand mosquitoes (Rattanarithikul et al. 2005, Rattanarithikul et al. 2007, Rattanarithikul et al. 2010, Jeffery et al. 2012).

### Statistical analyses

We first performed a linear correlation analysis to assess the relationship between species richness and species abundance to the total disturbance score. Following that, a regression analysis was performed to determine the significance of the relationship between species richness and species abundance to the total disturbance score. To assess how mosquito communities varied in each sampling site, we computed Bray-Curtis dissimilarity measured using square root transformed data. For visualisation of community composition, we produced a non-metric multidimensional scaling (NMDS) ordination plot. The Bray-Curtis dissimilarity measures were computed from the transformed data and subjected to NMDS to produce an ordination plot of mosquitoes. Sites that were clustered together in the ordination plots indicated that those sites were similar in mosquito composition. For rank abundance and community composition analyses, we use adult mosquito data as there was only one species of larval mosquito being collected in swamp forest, which was insufficient for these type of data analyses. All statistical analyses were computed using MS Excel ver. 16.16.3 (Microsoft Inc., USA), except for NMDS (GINGKO ver 1.5.8) (Bouxin 2005).

## Data resources

Raw data for adult mosquitoes are available in Table 2 and larval mosquitoes are available in Table 3.

## Results

### Disturbance scores

Four measures were used to quantify the disturbance at each sampling site and the values given for each measure were collected by two independent researchers (TPS and JML) and then summed up according to the sampling site, resulting in a total disturbance score (Table 1). The extent of soil profile disturbance was judged, based on disruptions, such as the introduction of pavements or walking paths and compactness to the soil are indications of high human activity in comparison to other sampling sites. Some of the sampling sites appeared to be disturbed over a longer period, such as the Chini Resort and the rubber plantation, while others displayed relatively recent disturbances, such as the hiking trail and the floating island. The frequency of disturbance also appeared to be higher at the site with the highest human activity, which is Chini Resort, while other sites with medium to low human activity displayed a low frequency or no disturbance. Overall, Chini Resort had the highest total disturbance score (8.0), followed by the rubber plantation (5.0), hiking trail (2.5) and the floating island (1.0). Based on the scale developed by Kimberling et al. (2001), we could not find any indication of human disturbance at the swamp forest site (0.0) (Table 1).

### Mosquito captures

Overall, a total of 661 adult mosquitoes, comprising 24 species (Table 2) and 411 mosquito larvae, comprising 16 species (Table 3) were sampled across five sampling sites within Chini Lake in July 2018. This includes commonly-known mosquito-borne disease vector genera, such as *Aedes*, *Anopheles*, *Culex* and *Mansonia*. Eleven species were captured as larvae that were not captured as adults were identified (Table 3). It was also discovered that all the adult species captured in Chini Resort were not mutually exclusive to that sampling site and occurred in three other sampling sites, with the floating island being the only exception.

Out of 411 mosquito larvae sampled, 323 were sampled from artificial breeding grounds, which consisted of plastic containers and plant pots, from the two sites with high disturbance scores, Chini resort and Rubber plantation. All larvae habitats at sites with low disturbance scores, the floating island and the swamp forest, were collected from natural breeding grounds, which consisted of water-filled tree holes and pitcher plants.

### Species richness, abundance and community

**Figure 2.**
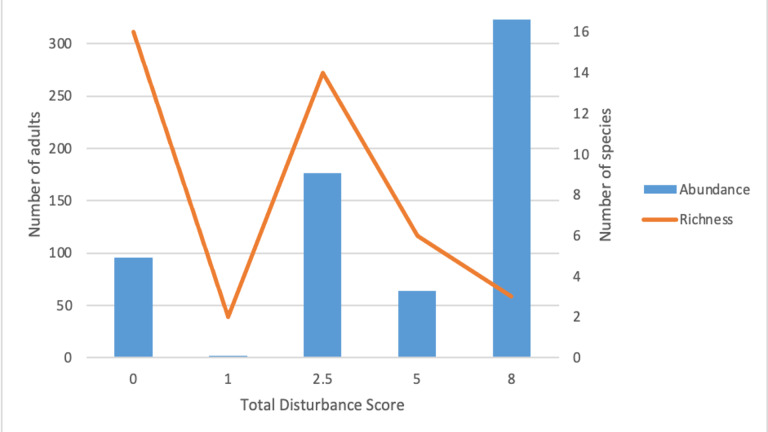
The number of adult mosquitoes and species collected across the anthropogenic disturbance gradients at Chini Lake surroundings. Species abundance (blue bars) increases at more anthropogenically disturbed sites. Conversely, species richness (orange lines) decreases at the more anthropogenically disturbed sites. Swamp forest (disturbance score = 0) has the highest number of species collected. An exception is seen at the floating island (disturbance score = 1), most likely due to the absence of large mammals at this sampling site.

**Figure 3.**
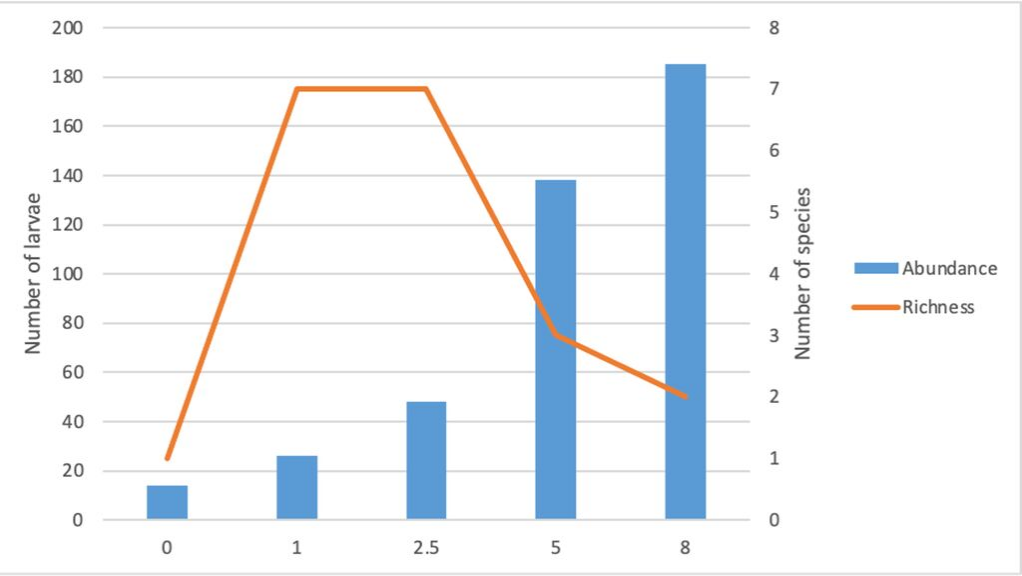
The number of mosquito larvae and species collected across the anthropogenic disturbance gradients at Chini Lake surroundings. Species abundance (blue bars) increases at more anthropogenically disturbed sites. Conversely, species richness (orange lines) decreases at the more anthropogenically disturbed sites.

Two genera dominated the total samples of adults and larvae collected; *Aedes* and *Culex* (*Table 3*). The mosquito community in Chini Lake consisted of a range of mosquito species, with 16 adult species recorded in swamp forest (disturbance score = 0), followed by the hiking trail (disturbance score = 2.5) with 14 species, the rubber plantation (disturbance score = 5.0) with six species, Chini Resort (disturbance score = 8) with three species and the floating island (disturbance score = 1.0) with two species (Figs 2, 3) Adult species richness displayed weak non-significant positive correlation to total disturbance score (R^2^ = 0.2849, *p* > 0.05, Fig. 2). Similarly, larval species richness also displayed weak non-significant positive correlation (R^2^ = 0.1010, *p* > 0.05, Fig. 3). Both adult and larval mosquito species richness showed a decreasing trend as the total disturbance score increases.

The highest number of adult and larval mosquitoes captured in a single sampling site were 325 and 185, respectively. Both of these values correspond to Chini Resort (disturbance score = 8.0). The lowest number of adult and larval mosquitoes captured in a single sampling site was 2 (floating island, disturbance score = 1.0) and 1 (swamp forest, disturbance score = 0.0), respectively (Table 3). In all but the floating island site, *Aedesalbopictus* dominated in terms of abundance of a single species at a given sampling site. Adult mosquito abundance showed a moderate non-significant positive correlation to total disturbance score (R^2^ = 0.5342, *p* > 0.05) (Fig. 2). Similarly, larval abundance showed a strong significant positive correlation to total disturbance score (R^2^ = 0.9795, *p* < 0.05) (Fig. 3). Both adult and larval mosquito abundance showed an increasing trend as the total disturbance score increases (Figs 2, 3). All sites, except the floating island (disturbance score = 1.0) have a highly uneven distribution of adult mosquitoes, with *Aedesalbopictus* dominating in these sampling sites (58.8% in Chini resort, 75.0% in Rubber plantation and 80.7% in Hiking trail).

**Figure 4.**
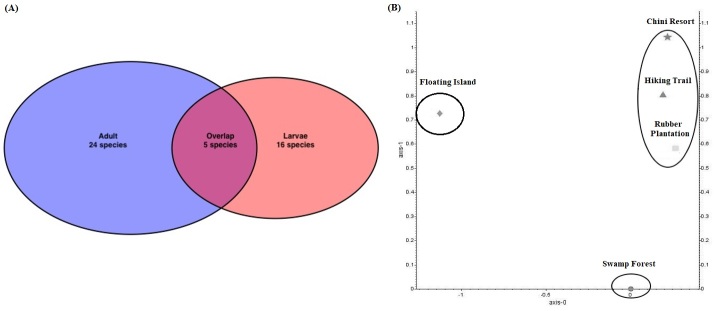
Mosquitoes captured between adult and larvae stages showed minimal overlap (A). Mosquito composition (both adults and larvae) at moderate to high-disturbed sites (disturbance score = 2.5 to 8) grouped, whereas the two low disturbance score sites differ from each other respectively (B).

Based on the Bray-Curtis dissimilarity measures, sites with high to moderate disturbance scores (2.5-8.0) were grouped in the NMDS plot. However, the two lowest disturbance score sites, swamp forest (disturbance score = 0.0) and floating island (disturbance score = 1.0) showed distinctively different mosquito community compositions (Fig. 4).

## Discussion

This study is the first to assess the effects of anthropogenic disturbance on mosquito species richness, abundance and community composition around Chini Lake. We used four semi-quantitative disturbance measures, which resulted in a total disturbance score for each of the five sampling sites (Table 1). These disturbance scores were biased towards the anthropogenic disturbance; for example, the extent of soil profile disturbance and the frequency of disturbance were scored highest at the site with the highest human activity - Chini Resort, while other sites with medium to low human activity displayed a low frequency or no disturbance. Overall, Chini Resort had the highest total disturbance score (8.0), followed by the rubber plantation (5.0), hiking trail (2.5) and the floating island (1.0). Based on the scale developed by Kimberling et al. (2001), we could not find any indications of human disturbance at the swamp forest site (0.0) (Table 1). At these sites, adult and larval mosquitoes were sampled twice and the species richness, abundances and community composition of mosquitoes at both life stages were analysed.

Mosquito species richness was higher in areas with lower anthropogenic disturbance (Figs 2, 3). This is consistent with mosquito diversity studies in the Neotropics (Loaiza et al. 2017, Loaiza et al. 2019), suggesting that a more disturbed environment is unable to accommodate mosquitoes with different ecological niches as opposed to a less disturbed environment (Steiger et al. 2016, Hermanns et al. 2021). This could be due to several reasons, one of which is that habitats with higher anthropogenic disturbances are associated with the introduction of artificial breeding grounds (Li et al. 2014, Tangena et al. 2016). Container breeders can exploit this breeding niche (Miyagi et al. 1992, Koenraadt and Harrington 2008, Li et al. 2014), allowing certain mosquito species that can tolerate feculent waters to thrive in conditions that are unfavourable for other mosquito species (Li et al. 2014, Zahouli et al. 2017a). This gives them an added advantage in anthropogenically disturbed environments over other mosquito species that can only breed under more stringent conditions (Vanwambeke et al. 2007). The mosquito species richness in less anthropogenically disturbed sites was mainly driven by natural breeding grounds’ availability, such as water-filled tree holes (Jenkins and Carpenter 1946). In these larval habitats, there is an abundance of aquatic insects that are natural predators to mosquito larvae (Quiroz-Martínez and Rodríguez-Castro 2007), which may control the over-dominance of a single mosquito species.

The second factor that may explain higher mosquito species richness in areas with lower anthropogenic disturbance is the change in landscape microclimate. Anthropogenically disturbed landscapes have more open habitats. The increased temperature and light intensity in open habitats can accelerate the growth and survivorship of mosquito larvae (Haider et al. 2017, Zahouli et al. 2017a, Zahouli et al. 2017b, Thomas et al. 2018). It also promotes algal growth (Steiger et al. 2016), an essential food source for mosquito larvae, directly contributing to the increased survivorship of larvae in these areas. This increased survivorship of some species in disturbed areas allows them to dominate the resources and persist in those areas as they occur in higher numbers (Loaiza et al. 2019), out-competing other less-tolerant species and consequently reducing species richness. Our sampling of larval mosquitoes at the disturbed sites collected 10x magnitudes of *Aedes* and *Culex* species in the two most anthropogenic disturbed sites (Table 3).

The third factor that may determine mosquito species richness is host availability and diversity (Minakawa et al. 2002, Smith et al. 2004, Burkett-Cadena et al. 2013). Female mosquitoes of almost all mosquito species require a blood meal from mammalian hosts to complete their reproductive cycle (Zhou et al. 2007). Large mammals are found on all sites, except the floating island. Due to its small size and inaccessibility (see Methods for site descriptions), adult female mosquitoes cannot thrive in that environment and consequently, adult male mosquitoes also do not inhabit as there is a lack of mating opportunities in the absence of female mosquitoes: hence, the lack of adult mosquito sampled on this site (Fig. 2). However, seven larvae species were identified in the floating island sampling site, suggesting that, although adult mosquitoes do not reside in that environment, the natural water holes in the ground and the pitchers of pitcher plants are suitable breeding grounds for mosquitoes (Fig. 3). We suspect that female mosquitoes use the site as breeding grounds and once the larvae emerge, they fly to nearby habitats for feeding (female) and mating (male) opportunities. Due to the contour of the Lake (Fig. 1), newly-emerged mosquitoes do not need to travel far for feeding and mating opportunities.

Surprisingly, we could not detect breeding grounds at our least disturbed site (= swamp forest) (Fig. 2). We suspected it is due to the month of sampling that coincides with the peak of the dry season. Natural larvae habitats in pristine environments are largely found to be reliant on rainfall (Tanaka and Tanaka 1982, Imbahale et al. 2011). This could have resulted in the low number of larval habitats found, which consequently led to the detection of low larvae species richness (Fig. 3). Although only one larvae species was identified, 16 species of adult mosquitoes were captured in the swamp forest (Fig. 2) and 15 of these species were distinct from the larvae species identified (Table 3). This indicated that adult mosquitoes can inhabit areas that are not suitable for larvae breeding, perhaps due to the availability of feeding and mating opportunities.

This study has a limited number of sites due to the inaccessibility of the areas surrounding Chini Lake. Moreover, the sampling time and capture method for adult mosquitoes were also biased towards capturing females. Due to the small replicates, we combined both sexes of mosquito in our analyses, excluding the analyses of female and male mosquito distribution differences. The aspiration method that we used for sampling targeted flying adults within the sampling radius could have very well missed those species that are not active during the sampling period. We include larvae mosquito sampling to partially rectify the adult sampling bias. This, however, introduces another sampling bias as not all species of mosquito are breeding simultaneously and the fact that some species would have emerged and had moved into an area from breeding sites far removed from the sampling radius. Nevertheless, we believed both adult and larvae mosquito sampling needs to be incorporated into all diversity surveys for taxa that have dual life stages, to understand a more comprehensive driver of diversity.

## Conclusions

A general pattern of reduced species richness, but increased species abundance was seen with increasing disturbance across the five sampling sites in Chini Lake, Pahang. Anthropogenic disturbances, especially in the form of deforestation and land-use change for agricultural and economic development lead to increased introduction of artificial breeding grounds and accessibility of mammal hosts for select mosquito species, such as *Aedesalbopictus* and *Culexbrevipalpis*. The inclusion of larval sampling is also highlighted as necessary, particularly for species not found in high numbers, potentially not active during crepuscular periods and species that use landscapes unlikely to be sampled by conventional approaches during the adult stage. Different resource requirements of adult and larval mosquitoes likely led to the observed diversity pattern in this small survey. We suggest future work to look into how habitat heterogeneity and availability of breeding grounds affect both sexes' mosquito diversity and distribution.

## Figures and Tables

**Table 1.** Disturbance scoring is based on Kimberling et al. (2001) scale. Chini Resort scores the highest in anthropogenic disturbance, whereas a site in the alluvial riparian swamp forest has no anthropogenic presence, hence scoring lowest on the disturbance scale.

Sampling site	Disturbance scores				
	Extent	Soil	Time	Frequency	Total
Chini Resort	2	2	2	2	8.0
Rubber plantation	1	0.5	1.5	1	5.0
Hiking trail	0.5	1.0	0.5	0.5	2.5
Floating island	0	0	1.0	0	1.0
Swamp forest	0	0	0	0	0.0

**Table 2.** Cumulative adult mosquito species were sampled across five sites with different disturbance scores (bracketed). Each site was sampled twice for 30 minutes respectively, once in the morning and once in the evening, within the periods of 7:00 AM to 8:00 AM, and 6:00 PM to 7:00 PM, coinciding with mosquito peak activity time.

Species	Sites & Disturbance Score (Highest to Lowest)				
	Chini resort (8)	Rubber plantation (5)	Hiking trail (2.5)	Floating island (1.0)	Swamp forest (0)
*Aedesalbopictus*	191	48	142	0	26
*Aedesbutleri*	0	0	0	0	19
*Anopheles* sp. 1	0	0	1	0	0
*Anopheleswhartoni*	0	1	10	0	1
*Armigeressubalbatus*	1	6	8	0	0
*Coquillettidianigrosignata*	0	2	3	0	3
*Coquillettidia* sp.	0	5	1	0	3
Culex (Lophoceraomyia) sp. 1	0	0	0	0	9
Culex (Lop.) sp. 2	0	0	0	0	3
*Culex* sp. 3	0	0	0	0	1
*Culexbrevipalpis*	133	0	1	0	6
*Culexcincetellus*	0	0	0	1	1
*Culexquinquefasciatus*	0	2	2	0	0
*Culexrubithoracis*	0	0	1	1	0
*Malaya* sp.	0	0	1	0	0
*Mansoniaannulata*	0	0	3	0	0
*Mansoniabonneae*	0	0	1	0	0
*Mansoniadives*	0	0	1	0	5
*Mansoniauniformis*	0	0	1	0	0
*Paraedesostentatio*	0	0	0	0	1
Tripteroides (Tripteroides) sp.	0	0	0	0	1
*Tripteroides* sp. 1	0	0	0	0	2
Uranotaenia (Uranotaenia) sp. 1	0	0	0	0	1
Uranotaenia (Ur.) sp. 2	0	0	0	0	14

**Table 3.** Larval mosquito species were sampled across five sites with different disturbance scores (bracketed). A sampling focal point was chosen at each site and larvae mosquitoes within the 100-m radius of the focal point were collected from both natural and artificial containers. '*' denotes larval mosquito species that were not captured as adults.

**Species**	**Sites & Disturbance Score Score (Highest to Lowest)**				
	Chini resort (8)	Rubber plantation (5)	Hiking trail (2.5)	Floating island (1.0)	Swamp forest (0)
*Aedesalbolineatus* *	0	0	11	0	0
*Aedesalbopictus*	146	119	1	0	0
*Aedespexa* *	0	0	14	0	0
*Aedesmikrokopian* *	0	0	9	0	0
*Anopheles* sp. 2 *	0	0	0	3	0
*Armigeresfollatus* *	0	0	1	0	0
*Culexrubithoracis*	0	0	0	15	0
Culex (Lop.) sp. 1	0	0	0	0	14
*Culexbrevipalpis*	39	0	7	0	0
*Mimomyia* sp. *	0	0	0	1	0
*Orthopodomyia* sp. *	0	0	0	1	0
*Toxorhynchitessplendens* *	0	2	0	0	0
Tripteroides (Rachionotomyia) sp. *	0	0	0	2	0
Tripteroides (Trp.) sp.	0	17	5	0	0
Uranotaenia (Pseudoficalbia) novobscura *	0	0	0	1	0
Uranotaenia (Ur.) micans *	0	0	0	3	0

## References

[B7842508] Afrane Yaw A., Lawson Bernard W., Yan Guiyun., Githeko Andrew K., Zhou Goufa (2006). Effects of microclimatic changes caused by deforestationon the survivorship and reproductive fitness of *Anophelesgambiae* in Western Kenya highlands. The American Journal of Tropical Medicine and Hygiene.

[B7842518] Ahmad Rohani, Ali Wan NWM, Nor Zurainee M, Ismail Zamree, Hadi Azahari A, Ibrahim Mohd N, Lim Lee H (2011). Mapping of mosquito breeding sites in malaria endemic areas in Pos Lenjang, Kuala Lipis, Pahang, Malaysia. Malaria Journal.

[B7776828] Burkett-Cadena Nathan D., McClure Christopher J. W., Estep Laura K., Eubanks Micky D. (2013). Hosts or habitats: What drives the spatial distribution of mosquitoes?. Ecosphere.

[B7842557] Haider Najmul, Kirkeby Carsten, Kristensen Birgit, Kjær Lene Jung, Sørensen Jens Havskov, Bødker Rene (2017). Microclimatic temperatures increase the potential for vector-borne disease transmission in the Scandinavian climate. Scientific Reports.

[B7854640] Hebauer F., Hendrich L., Balke M. (1999). A contribution to the knowledge of the water beetle fauna of a tropical freshwater lake: Tasek Cini, Pahang, West Malaysia (Coleoptera: Hydradephaga, Hydrophiloidea and Staphylinoidea).. *Raffles Bulletin of Zoology*.

[B7854834] Hermanns Kyra, Marklewitz Marco, Zirkel Florian, Kopp Anne, Kramer-Schadt Stephanie, Junglen Sandra (2021). Mosquito community composition shapes virus prevalence patterns along anthropogenic disturbance gradients. bioRxiv.

[B7842568] Imbahale Susan S, Paaijmans Krijn P, Mukabana Wolfgang R, van Lammeren Ron, Githeko Andrew K, Takken Willem (2011). A longitudinal study on Anopheles mosquito larval abundance in distinct geographical and environmental settings in western Kenya. Malaria Journal.

[B7842579] Jeffery J, Rohela M, Muslimin M, Abdul Aziz S, Jamaiah I, Kumar S, Tan T, Lim Y, Nissapatorn V, Abdul Aziz N (2012). Illustrated Keys: Some Mosquitoes of Peninsula Malaysia.

[B7776880] Jenkins Dale W., Carpenter Stanley J. (1946). Ecology of the Tree Hole Breeding Mosquitoes of Nearctic North America. Ecological Monographs.

[B7842603] Kaur G. (2009). Malaria endemicity in an Orang Asli community in Pahang, Malaysia. Trop Biomed.

[B7854662] Kemalok Jai, Mohamed Maryati, Rahman Aqilah AA, Ashikin Ismail Nurul (2019). Biodiversity across boundary: Ethnoentomology among the Jakun of Kampung Peta, Mersing and the Malay, Chinese and Indian of Kahang, Kluang, Johor. IOP Conference Series: Earth and Environmental Science.

[B7776917] Kimberling D, Karr J, Fore L (2001). Measuring human disturbance using terrestrial invertebrates in the shrub–steppe of eastern Washington (USA). Ecological Indicators.

[B7842623] Koenraadt C. J.M., Harrington L. C. (2008). Flushing Effect of Rain on Container-Inhabiting Mosquitoes<i>Aedes aegypti</i>and<i>Culex pipiens</i>(Diptera: Culicidae). Journal of Medical Entomology.

[B7842632] Lafferty Kevin D. (2009). The ecology of climate change and infectious diseases. Ecology.

[B7842641] Lam S., Chua K., Hooi P., Rahimah M., Kumari S., Tharmaratnam M., Chuah S., Smith D., Sampson I. (2001). Chikungunya infection - an emerging disease in Malaysia. Southeast Asian Journal of Tropical Medicine and Public Health.

[B7776935] Lee J. M., Wasserman R. J., Gan J. Y., Wilson R. F., Rahman S., Yek S. H. (2019). Human Activities Attract Harmful Mosquitoes in a Tropical Urban Landscape. EcoHealth.

[B7842548] Liang Guodong, Gao Xiaoyan, Gould Ernest A (2019). Factors responsible for the emergence of arboviruses; strategies, challenges and limitations for their control. Emerging Microbes & Infections.

[B7776955] Li Yiji, Kamara Fatmata, Zhou Guofa, Puthiyakunnon Santhosh, Li Chunyuan, Liu Yanxia, Zhou Yanhe, Yao Lijie, Yan Guiyun, Chen Xiao-Guang (2014). Urbanization Increases Aedesalbopictus Larval Habitats and Accelerates Mosquito Development and Survivorship. PLoS Neglected Tropical Diseases.

[B7776970] Loaiza Jose R., Dutari Larissa C., Rovira Jose R., Sanjur Oris I., Laporta Gabriel Z., Pecor James, Foley Desmond H., Eastwood Gillian, Kramer Laura D., Radtke Meghan, Pongsiri Montira (2017). Disturbance and mosquito diversity in the lowland tropical rainforest of central Panama. Scientific Reports.

[B7776986] Loaiza Jose R., Rovira Jose R., Sanjur Oris I., Zepeda Jesus Altagracia, Pecor James E., Foley Desmond H., Dutari Larissa, Radtke Meghan, Pongsiri Montira J., Molinar Octavio Smith, Laporta Gabriel Z. (2019). Forest disturbance and vector transmitted diseases in the lowland tropical rainforest of central Panama. Tropical Medicine & International Health.

[B7842688] Minakawa Noboru, Seda Pamela, Yan Guiyun (2002). Influence of host and larval habitat distribution on the abundance of African malaria vectors in western Kenya.. The American Journal of Tropical Medicine and Hygiene.

[B7842714] Miyagi Ichiro, Toma Takako, Mogi Motoyoshi (1992). Biological control of container-breeding mosquitoes, Aedesalbopictus and Culexquinquefasciatus, in a Japanese island by release of Toxorhynchitessplendens adults. Medical and Veterinary Entomology.

[B7842731] Mohd-Zaki Abdul Hamid, Brett Jeremy, Ismail Ellyana, L'Azou Maïna (2014). Epidemiology of dengue disease in Malaysia (2000-2012): a systematic literature review.. PLoS neglected tropical diseases.

[B7842754] Norris Douglas E. (2004). Mosquito-borne Diseases as a Consequence of Land Use Change. EcoHealth.

[B7842763] Olson Sarah H., Gangnon Ronald, Silveira Guilherme Abbad, Patz Jonathan A. (2010). Deforestation and Malaria in Mâncio Lima County, Brazil. Emerging Infectious Diseases.

[B7854616] Plan D. T. N. M. (1987). Department of Wildlife and National Parks..

[B7777024] Quiroz-Martínez Humberto, Rodríguez-Castro Ariadna (2007). AQUATIC INSECTS AS PREDATORS OF MOSQUITO LARVAE. Journal of the American Mosquito Control Association.

[B7842782] Rao B Bhaskar (2010). Larval habitats of Aedesalbopictus (Skuse) in rural areas of Calicut, Kerala, India.. Journal of vector borne diseases.

[B7777033] Rattanarithikul Rampa, Harrison Bruce A, Panthusiri Prachong, Coleman Russell E (2005). Illustrated keys to the mosquitoes of Thailand I. Background; geographic distribution; lists of genera, subgenera, and species; and a key to the genera.. The Southeast Asian journal of tropical medicine and public health.

[B7842791] Rattanarithikul R., Harbach R. E., Harrison B. A., Panthusiri P., Coleman R. E. (2007). Illustrated keys to the mosquitoes of Thailand V. Genera Orthopodomyia, Kimia, Malaya, Topomyia, Tripteroides and Toxorhychites.. The Southeast Asian journal of tropical medicine and public health.

[B7842807] Rattanarithikul R., Harbach R. E., Harrison B. A., Panthusiri P., Coleman R. E., Richardson J. H. (2010). Illustrated keys to the mosquitoes of Thailand. VI. Tribe Aedini. The Southeast Asian journal of tropical medicine and public health.

[B7842818] Shafie Aziz, Roslan Muhammad Aidil, Ngui Romano, Lim Yvonne Ai Lian, Sulaiman Wan Yusoff Wan (2016). Mosquito Biology and Mosquito-Borne Disease Awareness Among Island Communities In Malaysia. Journal of the American Mosquito Control Association.

[B7854597] Sharip Zati, Jusoh Juhaimi (2010). Integrated lake basin management and its importance for Lake Chini and other lakes in Malaysia. Lakes & Reservoirs: Science, Policy and Management for Sustainable Use.

[B7842828] Smith David L, Dushoff Jonathan, McKenzie F. Ellis (2004). The Risk of a Mosquito-Borne Infectionin a Heterogeneous Environment. PLoS Biology.

[B7854825] Steiger Dagmar Meyer, Ritchie Scott, Laurance Susan (2016). Mosquito communities and disease risk influenced by land use change and seasonality in the Australian tropics. Figshare.

[B7854650] Sujaul I. M., Ismail B. S., Muhammad B. G., Mohd E. T., Sahibin A. R. (2010). Assessment of land use and land cover changes in the Tasik Chini Catchment area, Pahang, Malaysia using the GIS.. *Advances in Environmental Biology*.

[B7854672] Syafiq Mustafa Muhammad Khairul, Sulaiman Junaida (2021). Dengue Dashboard for Forecasting the Future Trend of Dengue Cases in Pahang. 2021 International Conference on Software Engineering & Computer Systems and 4th International Conference on Computational Science and Information Management (ICSECS-ICOCSIM).

[B7842846] Tanaka L. K., Tanaka S. K. (1982). Rainfall and Seasonal Changes in Arthropod Abundance on a Tropical Oceanic Island. Biotropica.

[B7842855] Tangena Julie-Anne A., Thammavong Phoutmany, Wilson Anne L., Brey Paul T., Lindsay Steve W. (2016). Risk and Control of Mosquito-Borne Diseases in Southeast Asian Rubber Plantations. Trends in Parasitology.

[B7842865] Thomas Shalu, Ravishankaran Sangamithra, Justin N. A. Johnson Amala, Asokan Aswin, Kalsingh T. Maria Jusler, Mathai Manu Thomas, Valecha Neena, Montgomery Jacqui, Thomas Matthew B., Eapen Alex (2018). Microclimate variables of the ambient environment deliver the actual estimates of the extrinsic incubation period of Plasmodium vivax and Plasmodium falciparum: a study from a malaria-endemic urban setting, Chennai in India. Malaria Journal.

[B7842901] Vanwambeke Sophie O., Lambin Eric F., Eichhorn Markus P., Flasse Stéphane P., Harbach Ralph E., Oskam Linda, Somboon Pradya, van Beers Stella, van Benthem Birgit H. B., Walton Cathy, Butlin Roger K. (2007). Impact of Land-use Change on Dengue and Malaria in Northern Thailand. EcoHealth.

[B7842917] Vasconcelos Pedro F. C., Travassos da Rosa Amélia P. A., Rodrigues Sueli G., Travassos da Rosa Elizabeth S., Dégallier Nicolas, Travassos da Rosa Jorge F. S. (2001). Inadequate management of natural ecosystem in the Brazilian Amazon region results in the emergence and reemergence of arboviruses. Cadernos de Saúde Pública.

[B7777080] Zahouli Julien B. Z., Koudou Benjamin G., Müller Pie, Malone David, Tano Yao, Utzinger Jürg (2017). Urbanization is a main driver for the larval ecology of Aedes mosquitoes in arbovirus-endemic settings in south-eastern Côte d'Ivoire. PLOS Neglected Tropical Diseases.

[B7842946] Zahouli Julien B. Z., Koudou Benjamin G., Müller Pie, Malone David, Tano Yao, Utzinger Jürg (2017). Effect of land-use changes on the abundance, distribution, and host-seeking behavior of Aedes arbovirus vectors in oil palm-dominated landscapes, southeastern Côte d’Ivoire. PLOS ONE.

[B7842957] Zhou Guoli, Kohlhepp Pete, Geiser Dawn, Frasquillo Maria del Carmen, Vazquez-Moreno Luz, Winzerling Joy J. (2007). Fate of blood meal iron in mosquitoes. Journal of Insect Physiology.

